# The Modern Turkish Bath

**Published:** 1877-02

**Authors:** John Stainback Wilson

**Affiliations:** Atlanta, Ga.


### A BRIEF SKETCH OF THE HISTORY, NATURE, ADVANTAGES, AND PROCESSES OF THE MODERN TURKISH BATH.

By JOHN STAINBACK WILSON, M.D., Atlanta, Ga.

Some form of what is now called the Turkish bath existed among the Greeks, Romans, and other nations as far back as history extends. But though used by the people of ancient times, and by the modern Turks, as a luxury, and though it doubtless had much to do with the remarkable health and longevity of those who resorted to it, its scientific remedial use on rational principles, and in an improved form, may be properly dated only about twenty years ago, when it was introduced into Great Britain by Mr. David Urquhart.

Since that time its use has rapidly extended in Europe and in our own country; and after being tested in some of the largest hospitals and health institutions, thoroughly discussed in popular and professional works, by the best medical writers of the age, it has been decided in the light of modern science and experience to be a therapeutic agent unequalled in the correctness of its principles of application; in safety; in efficacy; and in the wide range of its usefulness.

This is doubtless true of the modern improved Turkish bath, as used among us, which excludes the coffee and the pipe of the Turks; in which the violent and unscientific shampooing and joint-cracking are not practised; and above all, where the genuine hot air bath is used in dry rooms, free from the slopping and steam of the bath as it is reported to be used by the Turks.

Now, as the difference between the steam and hot-air baths, and the decided advantages of the latter may not have been duly appreciated and considered, this article will be devoted mainly to a description of the nature, advantages, and processes of the improved modern Turkish bath.

As just intimated, this bath is not a steam bath, as many seem to think, but a *hot-air* bath. Steam is used in some baths for the purpose of heating steam coils or pipes from which the heat is radiated; but, so far as I am informed, most establishments in this country use and prefer dry furnace heat, which is diffused through the rooms by radiation and covection. But whether the heat be transmitted through steam pipes or radiated from heated surfaces, no steam is ever visible in a real Turkish bath; the object being to keep the air dry, for reasons that will be readily understood by physiologists.

In the genuine Turkish bath this hot air comes in contact with the whole surface of the body, including the head and lungs. No bath in which the body alone is included, leaving the head out, should be called a Turkish bath. The objections to these partial baths are so obvious that it is only necessary to mention them. These baths are deficient in all the essential features of a Turkish bath. One of these is to have rooms of *different temperatures*, so that the bather can go into a room moderately heated, and thus solicit a gentle general perspiration before going into a room of higher temperature, where the heat would be oppressive, if not dangerous, without having gone through the preparatory process of a room moderately hot. This preparation is essential to the safety and comfort of those who are not in the habit of bathing, and is an indispensable prerequisite to the use of that high heat on which the curative powers of the Turkish bath mainly depend.

Another essential feature of the Turkish bath is *breathing the warm air*. It is well known that the air-cells of the lungs are ten times more extensive than the whole surface of the body; and therefore, it can readily be seen how great are the advantages of a bath where an area of 20,000 square inches is brought in contact with warm, soothing, and highly electrified air, instead of having these cells exposed to an air of comparatively low temperature while the body is super-heated, as in the partial baths with the head out. And right here let me meet some of the objections that have been urged against breathing the warm air of a Turkish bath. One distinguished physician suggested to me that it would be likely to cause pneumonia. The answer to this is, that my own ample experience, and that of others, have fully demonstrated the fact that, so far from having an irritating action on the lungs, this air has a most delightful soothing effect, allaying cough, facilitating expectoration, and exerting the most decided and happy curative action in all pulmonary affections. Its effects in such cases are much like those of a warm poultice to an inflamed surface. I have often noticed that persons with the most harrassing cough, even in cases of advanced consumption, invariably cease coughing and breathe easier when in the hot air of my Turkish bath. These are the facts; and the physiological explanation does not seem difficult. Another objection urged by some against breathing the air of a Turkish bath is its assumed impurity. But, when the rooms of a Turkish bath are of the proper size, and ventilated as they can be, and are, in every well-constructed bath, the air does not become impure to any injurious extent. Certain it is, that breathing the air of a properly constructed Turkish bath is not so objectionable as being stewed in the confined and concentrated emanations of one's own body in any box or blanket bath, with the mistaken advantages of having the head out.

Annother essential point in the Turkish bath is wanting in the partial box or blanket bath, whether the bath be steam or hot air. As I may show at some future time, the shampooing is a most important part of a Turkish bath; and to do it effectually it must be done in a room *heated to a high temperature;* which cannot be, unless a room is provided for the purpose, as it is in every properly constructed Turkish bath.

Now, a few words as to the advantages of a hot-air or Turkish bath over a steam bath, or any other kind of bath. The objections to box baths already mentioned, o course apply equally to vapor as to hot air when thus partially administered; this steam box bath being only the old Thompsonian bath with which all are acquainted. But some may think that the so-called Russian bath, or the Turco-Russian bath, is preferable to the Turkish or hot-air bath. True, we have establishments well fitted up, where bathers are put, head and body, into a room filled with vapor, and then this general vapor bath is followed by a shampooing, washing with water, etc., as in the Turkish bath. Without the shampooing, it is called simply the Russian bath; when the shampooing is added, it is called the *Turco*-Russian bath.

This is certainly a better bath than any box bath, whether the contents of the box be hot air or steam. But, as we shall see, this form of bath is still deficient in every important element which gives to the Turkish bath its great superiority over every other.

In the first place, these baths are not so constructed as to afford those graded and gradually increased temperatures that are so perfectly obtained in the separate rooms of a Turkish bath heated to different degrees. The Russian or vapor bath generally consists of but one room; and the only difference of temperature is to be had by changing places in this single room. But the great trouble with these baths is the *difficulty in breathing* vapor of a high temperature, or indeed of any temperature. While, in a Turkish bath, the air can be breathed with positive delight to the very bottom of the lungs, at a temperature of from 150° to 200°, and even higher; vapor heated from 110° to 120° is suffocating and anything but pleasant to inhale. We all know when the air at ordinary temperatures is loaded with moisture that, with few exceptions, the respiration is oppressed, and there is general dullness and *malaise.* The cause of this is too well known to need explanation; and this cause, existing in the highest degree in the air of a room surcharged with vapor, it is manifest that most of the advantages of inhalation alluded to in connection with the hot air of the Turkish bath, are lost in the vapor bath.

Again, the vapor bath not only interferes with pulmonary respiration, but it is far less promotive of cutaneous transpiration than the hot-air bath. Indeed, this vapor is an obstacle to perspiration. This transpiration is of course proportioned to the increased temperature of the medium by which we are surrounded, to the dryness, and also to the rarity of the medium—a circumstance scarcely less favorable to transpiration than a high temperature. And, as in the Turkish bath, we have a much higher temperature than can be endured or obtained in any other; and as with this we have air of great levity and with but little moisture, it is plain that this bath is far superior to the vapor bath or any other, as a means of exciting the capillary circulation and the transpiration of the skin. This, together with the action of the warm air on the lungs, which may be explained more fully hereafter, leaves the Turkish bath without a rival in the way of baths, and makes it the perfection of this class of remedies; and as perfection can never be excelled, the Turkish bath will always be acknowledged as superior to all other baths by those who understand the plain philosophy on which it is founded; and especially by those who know this philosphy to bé true as demonstrated by the results of experience in the perfect restoration of a large class of patients incurable by other means. But some may say that in dry, scaly skin diseases, in herpetic affections, and perhaps in some other cases, the vapor bath would be preferable. This may be so in some cases, but the number will be small; for, in the Turkish bath the patient always supplies a sufficient quantity of water from his own person to soften the skin thoroughly.

If any more is needed it can very readily be obtained by wrapping the body in a warm wet sheet while in the bath, thus extemporising a vapor bath without its objections, and at the same time combining the advantages of the hot-air bath, and the next best bath to it—the wet-sheet pack.

PROCESSES OF THE TURKISH BATH.

After what has been said as to the essential features of a Turkish bath, such as its rooms of different temperatures; its light, dry, warm air, applied to the whole surface of the body and the extensive cellular structure of the lungs; and its hot room for shampooing, a pretty good idea may be formed as to the parts and processes of this bath. But a brief description of these may be given.

Every well constructed Turkish bath should have not less than four large, well ventilated rooms. The first is the *Frigidarium,* or cooling room, part of which is used as dressing rooms. The second is the *Tepidarium,* or first warm or tepid room. The third is the *Calidarium,* or hot room. And the fourth is the *Lavatorium,* or wash room. The bathing rooms proper, in my bath, that is, the Tepidarium, the Calidarium, and the Lavatorium, are 14x16 feet in width and length, and 9 feet high, giving over 2000 cubic feet of air to each. The two hot-air rooms are provided each with a ventilator 12x8 inches, running from windows on the outside of the rooms and beneath the floors, with the internal openings of these ventilators entering the rooms near the heating apparatus; thus making a direct current of pure air from all *out-doors,* which can be let in at any time, sweeping out every impurity in a few minutes, through the flue and through openings, provided for this purpose in the upper part of the rooms. Thus is the purity of the air secured both by the size of the rooms and by a sufficiently free communication with the whole ocean of external atmosphere; and thus is all danger of impurity guarded against.

Let us now briefly notice the *processes* of a Turkish bath.

The bather first enters the frigidarium, where he undresses and passes into the tepidarium. The temperature of this ranges from 110° to 140° the object being to get up a general perspiration, without any undue excitement or oppression, before going into the next or hot room. To accomplish this end more effectually, and to guard against any undue determination to the brain or other internal organ until the circulation is diffused throughout the whole cutaneous surface, it is my practice to apply a cold wet cap to the head, at the same time using a hot foot-bath, having this to come well up the legs. By this course all trouble arising from irregular distribution of blood is effectually obviated, and perspiration is excited without the least feeling of oppression or excitement in almost every case; and should there be a slight feeling of oppression, it is only momentary, passing off after the first few inspirations. When a general moisture appears on the skin, the bather is then passed into the calidarium, hot, or sweating room, which is heated from 140° to 180° or 200°. Here the shampooing is done while the bather furnishes the water from his own skin, and while every thing on the surface, and many things from *within*, are all *afloat*. In some establishments the shampooing is done in a room of much lower temperature than the calidarium; but I find it much better to perform this important part of the process in a high temperature; which is more pleasant to the bather, and much more effective, as any one will readily see. Indeed, this shampooing in the hot room is not only one of the most useful, but the most delightful of all the processes of the Turkish bath, as every bather will testify. The feeling of delightful repose of body and mind under this high heat and friction is indiscribable, and can never be fully appreciated until enjoyed.

As to the shampooing, a few words of description will suffice. It consists in a thorough rubbing of the whole surface of the body with the *hands* of the attendant, and not with coarse, rough cloths, horse-brushes, or brick-bats, as some seem to imagine. All the rubbing and scrubbing, pulling, scraping, baking and roasting described by some, exist only in imagination, and are no parts of a well conducted, scientific Turkish bath. When the shampooing is done in the hot room as I contend it should be, nothing more is necessary in the way of thorough cleansing and a perfect capillary circulation than a good rubbing with the hands, followed by percussion or slapping over the surface, and then by a moderately coarse cloth and good soap. This does the work fully, and leaves the bather with plenty of skin for all the purposes for which this investment was designed; at the same time greatly facilitating its most important functions.

Except in some rare cases, neither flesh brushes nor coarse, rough cloths should be used in the Turkish bath; because they are useless, and might, in some cases, be injurious.

Having now sweated, shampooed, percussed, and soaped our bather, we will now put him into the lavatorium or wash room. Here we have a temperature approaching 100°, but of course it feels much cooler after passing from a room heated to 160°. Still he is not likely to complain of the transition, which is rather pleasant than otherwise.

Getting him into this room, he next takes a warm shower or spray bath; and the temperature of this is gradually reduced until the water is quite cool or cold. The object of this is not only to cleanse him, but to close the pores of the skin, and cause a vigorous reaction, which is very readily done after passing through the hot room, which has caused such a strong determination to the skin. After this the feeblest patients generally react without difficulty.

Many bathing establishments use the plunge bath after passing the bather through the hot room. But I object to this as a needless shock, which is unpleasant, and to say the least of it, is unnecessary. As the hands will accomplish all that is necessary in shampooing, so will the shower or spray bath accomplish all that is needful or desirable in the way of washing, closing the pores, cleansing perfectly, and causing a strong reaction.

Having now carried our bather through all the rooms, and submitted him to all the processes of the Turkish bath, he returns to the frigidarium or cooling room, where he is directed to dress slowly without further delay, or if feeble or disposed to break out into a secondary perspiration, he is wrapped in a sheet or blanket, reclines on a lounge, and remains there in an air bath of the ordinary temperature, until he is "*all right.*" He then dresses and goes out, reinvigorated in mind and in body, with energies renewed, ready to do or dare anything in the compass of human power; and not only cleaner than ever before, but in every way a better man, in mind, body, and spirit.

